# Inter-annual decrease in pulse rate and peak frequency of Southeast Pacific blue whale song types

**DOI:** 10.1038/s41598-020-64613-0

**Published:** 2020-05-15

**Authors:** Franck Malige, Julie Patris, Susannah J. Buchan, Kathleen M. Stafford, Fannie Shabangu, Ken Findlay, Rodrigo Hucke-Gaete, Sergio Neira, Christopher W. Clark, Hervé Glotin

**Affiliations:** 1 Univ. Toulon, Aix Marseille Univ., CNRS, LIS, DYNI team, SABIOD, Marseille, France; 2 COPAS Sur-Austral, Edificio Departamento de Oceanografía Piso 2 Barrio Universitario s/n, Universidad de Concepción, Concepción, Chile; 3 Centro de Estudios Avanzados en Zonas Áridas, Avenida Ossandón 877, Coquimbo, Región de Coquimbo, Chile; 4 Woods Hole Oceanographic Institution, Biology Department, Woods Hole, Massachusetts, 02543 USA; 5 Applied Physics Laboratory, University of Washington, 1013 NE 40th Street, Box 355640, Seattle, Washington 98105 USA; 6 Fisheries Management, Department of Agriculture, Forestry and Fisheries, Private Bag X2, Vlaeberg, Cape Town, 8012 South Africa; 7 Mammal Research Institute Whale Unit, University of Pretoria, Private Bag X20, Hatfield, Pretoria 0028 South Africa; 8 Cape Peninsula University of Technology, P.O. Box 652, Cape Town, 8000 South Africa; 9 Instituto de Ciencias Marinas y Limnologicas, Universidad Austral de Chile, Campus Isla Teja, Valdivia, Chile; 10 Bioacoustics Research Program, Cornell Laboratory of Ornithology, Cornell University, Ithaca, New York 14850 USA

**Keywords:** Marine biology, Acoustics

## Abstract

A decrease in the frequency of two southeast Pacific blue whale song types was examined over decades, using acoustic data from several different sources in the eastern Pacific Ocean ranging between the Equator and Chilean Patagonia. The pulse rate of the song units as well as their peak frequency were measured using two different methods (summed auto-correlation and Fourier transform). The sources of error associated with each measurement were assessed. There was a linear decline in both parameters for the more common song type (southeast Pacific song type n.2) between 1997 to 2017. An abbreviated analysis, also showed a frequency decline in the scarcer southeast Pacific song type n.1 between 1970 to 2014, revealing that both song types are declining at similar rates. We discussed the use of measuring both pulse rate and peak frequency to examine the frequency decline. Finally, a comparison of the rates of frequency decline with other song types reported in the literature and a discussion on the reasons of the frequency shift are presented.

## Introduction

Blue whale (*Balaenoptera musculus*) songs are the repetition of several highly stereotyped low-frequency, high energy units that compose song phrases, first described in 1971^1^. Song units and phrases have been qualified as‘remarkably consistent’ within a song, but also between individuals^1^. Song in blue whales has been attributed to reproductive display by males^2^. Numerous, distinct songs have been identified worldwide^3^, each displaying stability in the temporal and frequency characteristics of units and phrases and intervals between units or phrases. However, this global pattern has been shown to be affected by a general decreasing trend in frequency over decadal timescales^4^.

This linear decline in tonal frequencies of blue whale song types is a recently described unexplained phenomenon. It appears to occur worldwide, based on analyses of different regional song types, spanning five decades^4^. New studies have recently confirmed these results for Antarctic blue whale song type^5–7^ for the southwestern Pacific Ocean song type^8^, or for different song types in the Indian Ocean^7,9,10^. So far, no studies have examined frequency shift in southeastern Pacific Ocean blue whale songs, even though these were the first blue whale songs to be identified as such^1^. A similar inter-annual frequency decrease has been recently measured for bowhead whale (*Balaena mysticetus*)^11^ and fin whale (*Balaenoptera physalus*) populations^7,12^ and in other low frequency sounds attributed to unidentified baleen whales^6,13^.

**Figure 1:**
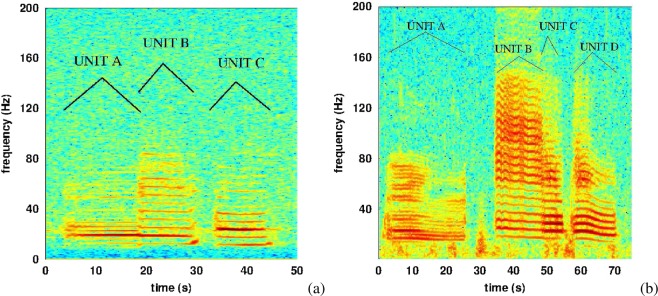
(**a**) Time/frequency representation of a phrase of the SEP1 song, recorded in the Corcovado Gulf, Chile, 2012 March 1st, sample rate *f*_*s*_ = 2 kHz, FFT 2^12^ points, overlap of 90%, Blackman window. (**b**) Time/frequency representation of a phrase of the SEP2 song, recorded off Isla Chañaral, Chile, 2017 February 2nd, sample rate *f*_*s*_ = 48 kHz, FFT 2^16^ points, overlap of 75%, Blackman window.

There are two blue whale song types in the southeast Pacific Ocean known as SEP1 and SEP2. SEP1 was first described almost fifty years ago^1^, while SEP2 was first recorded in 1996^14^ near the Equator and described in detail as a new song type in 2014^15,16^. In recent times, SEP2 has been found to be the dominant song type in the Chilean Patagonia^17,18^. These songs are each composed of a single repeated phrase, highly stereotyped in unit composition, duration and frequency characteristics (see Fig. 1). The SEP1 phrase is composed of 3 units^1^ called *A*, *B*, *C* and shown in Fig. 1, left, whilst the SEP2 phrase is composed of 4 units^16^ called *A*, *B*, *C* and *D* and shown in Fig. 1, right. The SEP1 and SEP2 phrases are usually repeated every 100 s and 120 s respectively (called inter-song interval, in the literature), in sequences lasting from some minutes to a few hours (called a song)^1,15^. All these features are observable only in clear recordings, with high signal to noise ratio (SNR). Units *C* and *D* of SEP2 are usually the loudest and together have been used as a kernel for automatic detection^17^.

**Figure 2:**
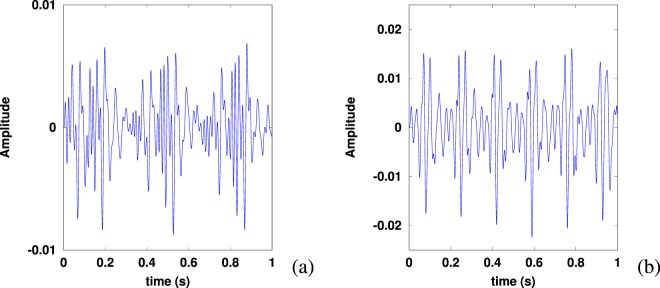
Extracts of units *A* (**a**) and *C* (**b**) of a SEP2 song recorded off Isla Chañaral, 2017 February 2nd, in waveform.

One of the defining characteristics of these song types is the pulsed nature of their units, visible in Fig. 2 as a repetition rate at low frequency. The SEP1 units have a pulse rate *f*_*pulse*_ around 6 pulses per second for units *B* and *C*, and 3 pulses per second for unit *A*. The SEP2 units have a pulse rate *f*_*pulse*_ around 6 pulses per second for units *B*, *C* and *D*, and 3 pulses per second for unit *A* (see data analysis section for measurement techniques). This pulse rate can also be seen as the frequency gap between two bands in Fig. 1^19^. In the case of SEP2 song type, the pulse rate is in fact the fundamental frequency of each song unit^20^, which is nevertheless not appearing in the spectrum, as seen in Fig. 1.

These song types have been recorded at different sites in the eastern Pacific Ocean: near the Equator, in the eastern tropical Pacific (ETP) between 1996 and 2002^14^, off the north coast of Chile in 1997–1998 by the International Whaling Commission’s Southern Ocean Whale and Ecosystem Research (IWC SOWER) program^21^, in the Corcovado Gulf in the south of Chile in 2012, 2013, 2016, 2017^17^, near the Juan Fernandez archipelago off Chile in 2005, from 2007 to 2010 and from 2014 to 2016 by the Comprehensive Nuclear-Test-Ban Treaty Organization (CTBTO https://ctbto.org/) and off Isla Chañaral, Chile, in 2017^22^.

In this paper, we examine the frequency characteristics of the SEP song types by computing the pulse rate and peak frequency of their units to determine whether frequency decline has occurred in the last decades, using seven different data sets. We also discuss the sources of error associated with both pulse rate and peak frequency measurements.

## Data collection

**Figure 3:**
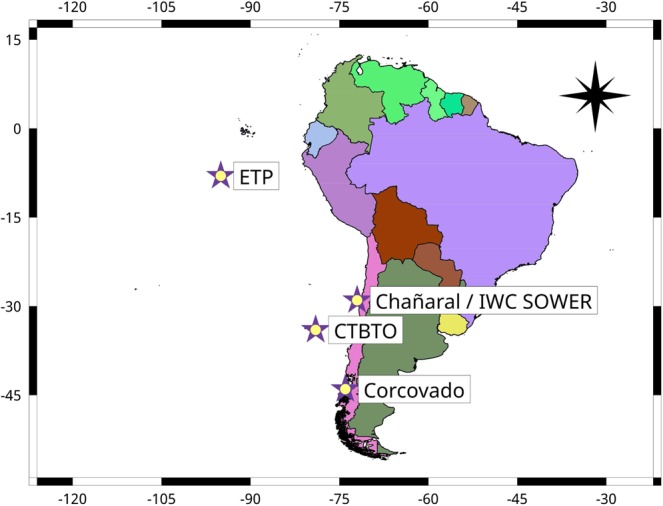
Location of the recording stations used in this study.

Because it is the predominant song type, we first analyzed in detail 436 SEP2 song phrases spanning 21 years (1996–2017), from 5 different locations and 7 different data sets listed in Table 1 and displayed in Fig. 3.

**Table 1:** Characteristics of the data analyzed. Table includes name of the data set, references, place, season, number of phrases analyzed and sample rate of the data.

Data Set	Latitude (S)/longitude (W)	Year	Austral season	Number of SEP1/2 phrases	Sampling rate *f*_*s*_ (Hz)
ETP 1 (a)	8°/95°	1996	winter	23/50	100
IWC SOWER (b)	29°/72°	1997	sum.	−/36	1000
ETP 2 (a)	8°/95°	2002	winter	−/50	250
CTBTO (c)	34°/79°	2007	fall	31/-	250
CTBTO (c)	34°/79°	2009	fall	−/50	250
Corcovado 1 (d)	44°/74°	2012	sum.	33/50	2000
Corcovado 1 (d)	44°/74°	2013	sum.	−/50	2000
CTBTO (c)	34°/79°	2014	sum.	20/50	250
Corcovado 2 (f)	44°/74°	2016	sum.	−/50	4000
Chañaral (e)	29°/72°	2017	sum.	−/50	48000

References: (a): Stafford *et al*.^14^, (b) Shabangu *et al*.^21^, (c) CTBTO https://ctbto.org/, (d) Buchan *et al*.^17^, (e) Patris^22^, (f): Buchan *et al*., unpublished.

Each year, files were browsed day by day to spot some high SNR sequence of SEP1 or SEP2. Fifty high SNR phrases were manually selected for each year. To minimize the probability of only analyzing phrases from a single song bout, phrases were selected from five different days. In the case of the SOWER data, only 36 phrases from three different days were selected, due to the short duration of the data set.

The oldest recordings for SEP2 is 1996. For the SEP1 song type, however, the first description was as far back as 1970^1^. We were not able to access the raw data for this recording, however we made use of the results published in the paper^1^, where only pulse rate, but not peak frequency is reported precisely. Over all available data bases, there are fewer examples of SEP1. With these limitations, we applied the same approach for SEP2 to SEP1 examples to compute peak frequency and pulse rate and examine whether SEP1 has the same rate of decrease as SEP2. The number, location and season of the SEP1 occurrences analyzed are displayed in Table 1. This allows us to examine SEP1 frequency shift over 44 years (1970–2014), which is double the time period available for SEP2, although with less data.

## Data analysis

### Methods

To estimate the frequency decrease, we measured the peak frequency and pulse rate of units of each phrase of SEP song types. This study is based on the theoretical description of pulsed sounds and on the measurement of their characteristics presented in^20^.

#### Fast Fourier transform (FFT) to measure the peak frequency

The spectral power density of whales’ vocalizations usually presents a set of discrete peaks. To measure the frequency trend of a song it is important to choose the peak consistently from one signal to other. Strictly speaking, the so-called “peak frequency” *f*_*peak*_ is the frequency of the peak with maximum energy. This definition has been used for instance in^16^. However, the peak where energy is maximum can be highly variable due to environmental effects or sensor sensitivity even though the general set of frequency peaks within a single unit is very stable at subdecadal timescales. Following^4,5,7^ we defined ‘peak frequency’ as the frequency of the band that is *on average* the one with maximum energy, which in this case is around 25 Hz for the SEP song types. When maximum energy is shifted to the 32 Hz frequency band, we still measured the exact frequency of the band around 25 Hz, in order to ensure a standard metric for examining the decadal trend in the song frequencies.

For all selected units, we performed a FFT on the first 4 s of the unit by a routine in OCTAVE^23^. We measured the peak frequency as the frequency corresponding to the maximum value (in modulus) of the FFT between 23 and 26 Hz for unit *A* of each song type and 22 and 27 Hz for the other units. Long term spectral averages^5^ were not computed because of the complex nature of SEP songs compared, for example, to the Antarctic blue whale song type as the frequencies of different parts of the song overlap, blurring the long-term average.

#### Auto-correlation of the signal to measure the pulse rate

For SEP2, units are periodic, or harmonic, signals: the pulse rate (*f*_*pulse*_) is the fundamental frequency of these units and the ratio $\frac{{f}_{peak}}{{f}_{pulse}}$ is an integer^20^. This ratio is equal to 8 for unit *A* and 4 for units *B*,*C* and *D*. For SEP1 units, we checked first that units *B* and *C* are harmonic following the method presented in^20^ (see section results below for details). In this case, the pulse rate can be accurately measured by a summed auto-correlation of the signal when the sample rate of the recording is high (see the following section, about associated uncertainties). The auto-correlation function of a signal *s* is

1 $$C(\tau )={li}{{m}}_{T\to +\infty }\frac{1}{T}{\int }_{-T/2}^{T/2}s(t){s}^{\ast }(t+\tau )dt$$

(where s* is the complex conjugate of *s*). In practice, *s* is a real signal, time *T* is limited by duration ${T}_{{\rm{s}}{\rm{i}}{\rm{g}}{\rm{n}}{\rm{a}}{\rm{l}}}$ of the unit and our signal is sampled at a frequency *f*_*s*_. We thus define an approximation of $C(\tau )$ by

2 $${C}_{{T}_{{\rm{signal}}},{T}_{s}}(\tau )=\mathop{\sum }\limits_{n=0}^{\lfloor {T}_{{\rm{signal}}}/{T}_{s}\rfloor }s(n{T}_{s})s(n{T}_{s}+\tau )$$

where ${T}_{s}=\frac{1}{{f}_{s}}$ is the sampling interval and $\lfloor x\rfloor$ is the integer part of a real number *x*. Here, we have ${T}_{{\rm{signal}}}=4$ s. If the signal *s* is harmonic, the function $C(\tau )$ has maximums when $\tau$ is a multiple of the period. For a description of auto-correlation techniques applied to mysticete sounds see for example^24^.

**Figure 4:**
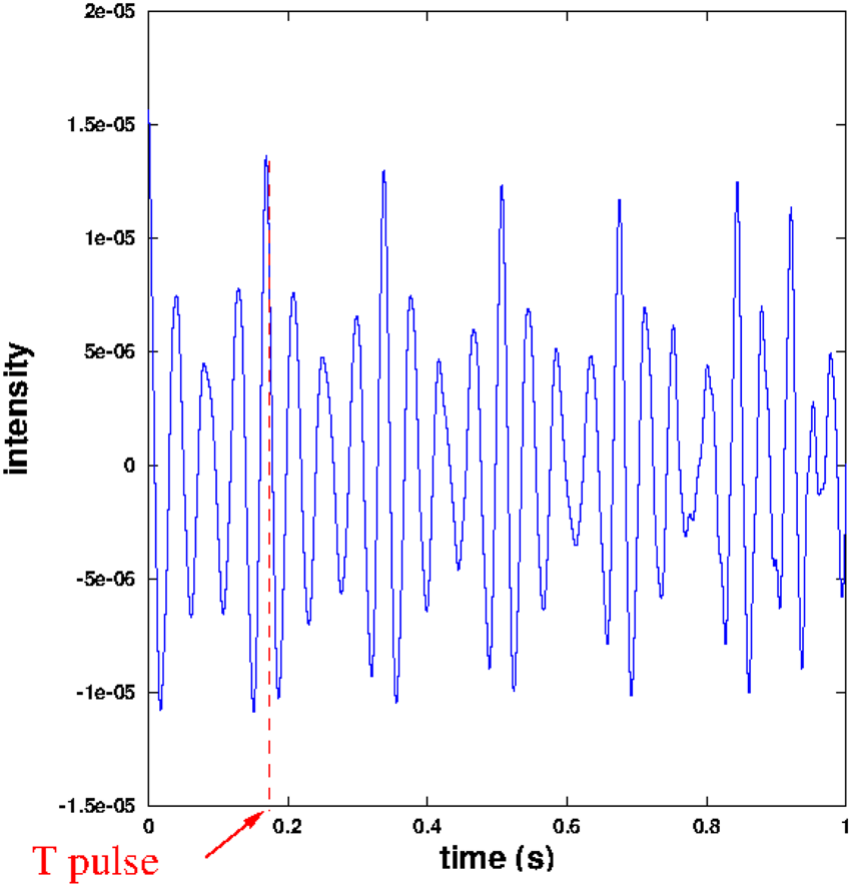
Graph of the auto-correlation ${C}_{{T}_{{\rm{signal}}},{T}_{s}}(\tau )$ of unit *C* of a SEP2 phrase: the maximum of correlation for ${T}_{pulse}\simeq 0.17$ s corresponds to a frequency ${f}_{pulse}\simeq 6$ Hz. The time ${T}_{{\rm{signal}}}$ is 4 s.

For each unit, we computed the approximate auto-correlation function (using an OCTAVE dedicated routine) for $\tau \in \mathrm{[0:}{t}_{correl}=1$ s] with a step of ${T}_{s}$ (see Fig. 4). A low-pass filter (fifth order Butterworth with frequency cut-off at 150 Hz) was applied before the auto-correlation to reduce high frequency noise. To obtain a maximum likelihood of the pulse frequency measurement, we performed a refinement of the auto-correlation method^25^, involving the computation of the summed auto-correlation function

3 $$g(t)=\frac{t}{{t}_{correl}}\mathop{\sum }\limits_{n=1}^{\lfloor {t}_{correl}/t\rfloor }{C}_{{T}_{{\rm{s}}{\rm{i}}{\rm{g}}{\rm{n}}{\rm{a}}{\rm{l}}},{T}_{s}}(nt)$$

We computed this function for all values of *t* between 0 and ${t}_{{\rm{correl}}}$ with a step ${T}_{s}$. This function has a peak for *t* equal to the period. We thus measured the corresponding time *T*_*pulse*_ of the highest peak of the summed auto-correlation in the interval $t\in \mathrm{[0.15;}\,\mathrm{0.175]}$ s (corresponding to frequencies between 5.71 and 6.66 Hz) which gives the frequency ${f}_{pulse}=\frac{1}{{T}_{pulse}}$.

#### Error in frequency estimation

Uncertainties arise from three main sources: The inherent error of each method (quantification error) in measuring *f*_*pulse*_ and *f*_*peak*_, ambient noise and the intrinsic dispersion in whale vocalizations. We assessed separately these three causes of uncertainty in the measurements.

#### Uncertainties due to the method: quantification errors

Measuring peak frequency by means of a FFT cannot be more precise than the frequency step of the FFT (quantification of the frequency). Given that useful parts of SEP units are around ${T}_{{\rm{signal}}}=$ 4 s long, the highest resolution FFT that can be computed is ${f}_{s}\times {T}_{{\rm{signal}}}$ points. This results in a precision in frequency of $\frac{{f}_{s}}{{f}_{s}\times {T}_{{\rm{signal}}}}=\frac{1}{4}=0.25$ Hz. We note that this uncertainty is $\mathrm{1/}{T}_{{\rm{signal}}}$ and thus does not depend on the sampling rate *f*_*s*_.

On the other hand, the quantification error of the summed auto-correlation method depends strongly on *f*_*s*_. To estimate ${f}_{pulse}$, assuming that the recording device has a sample rate *f*_*s*_, the uncertainty in time $\Delta {T}_{pulse}$ when we measure ${T}_{pulse}$ is thus at least of the order of $\mathrm{1/}{f}_{s}$. We have ${f}_{pulse}=\mathrm{1/}{T}_{pulse}$ so the corresponding uncertainty in frequency $\Delta {f}_{pulse}=\Delta {T}_{pulse}/{T}_{pulse}^{2}={f}_{pulse}^{2}\Delta {T}_{pulse}\simeq {f}_{pulse}^{2}/{f}_{s}$. In the case of a sample rate of 48 kHz and for a measured frequency ${f}_{pulse}$ around 6 Hz, the quantification error is $\Delta {f}_{pulse}\simeq {10}^{-3}$ Hz. But for ${f}_{s}=2$ kHz, we have $\Delta {f}_{pulse}\simeq 0.05$ Hz and for ${f}_{s}=100$ Hz, we have $\Delta {f}_{pulse}\simeq 0.2$ Hz.

We can also compute (and compare) the relative quantification error $\varDelta f/f$. For our signals, we usually have ${f}_{pulse}\simeq 6$ Hz and ${f}_{peak}\simeq$ 24 Hz. The relative error of ${f}_{peak}$ measurement is thus $\Delta {f}_{peak}/{f}_{peak}\mathrm{=1/(}{T}_{signal}\times {f}_{peak})\simeq$ 1% for each recording. The relative error of *f*_*pulse*_ measurement depends on the sample rate: $\Delta {f}_{pulse}/{f}_{pulse}={f}_{pulse}/{f}_{s}$. It becomes smaller than 1% for *f*_*s*_ greater than 400 Hz (${f}_{s}\mathrm{=100}$ Hz leads to $\Delta {f}_{pulse}/{f}_{pulse}\simeq$ 6%, ${f}_{s}\mathrm{=2000}$ Hz leads to $\Delta {f}_{pulse}/{f}_{pulse}\simeq$ 0.3%). Thus, pulse rate measurement has a smaller systematic relative uncertainty than peak frequency measurement for *f*_*s*_ greater than 400 Hz.

#### Uncertainty due to noise

To compute SNR, the following approach was used: The energy of a unit of duration ${T}_{{\rm{s}}ignal}$ is proportional to ${\int }_{0}^{T\text{signal}}$|*s*(*t*)|^2^*dt* ^26^. We computed an approximation of the energy (where the coefficient *A* depends on the sampling rate but is constant for a given signal and will not appear in the SNR):

$${E}_{{\rm{SEP}}2}=A\mathop{\sum }\limits_{n=0}^{\lfloor {T}_{{\rm{signal}}}/{T}_{s}\rfloor }|s(n{T}_{s}){|}^{2}$$

We measured the energy of each unit by computing the energy *E* of the signal during 4 s. A band-pass filter (fifth order Butterworth with frequency band 5–50 Hz) was applied before the computation of energy. We then calculated the SNR by estimating the energy ${E}_{N}$ of the background noise at a time selected manually before or after each SEP2 phrase, using the same formula and during the same ${T}_{{\rm{s}}{\rm{i}}{\rm{g}}{\rm{n}}{\rm{a}}{\rm{l}}}$. We compute $SN{R}_{(dB)}\mathrm{=10}{\log }_{10}(\frac{{E}_{{\rm{SEP}}2}}{{E}_{N}})$ for each SEP2 song phrase. The SNR varied from 1 dB to 40 dB in the 436 selected SEP2 phrases.

To check each of our method’s resistance to background noise, we selected one song from 2012 February 14th in Corcovado with high SNR (around 40 dB) and we added background noise (taken from the same recording) with increasing levels, resulting in a deterioration of the SNR. We then measured the peak frequency and pulse rate.

**Figure 5:**
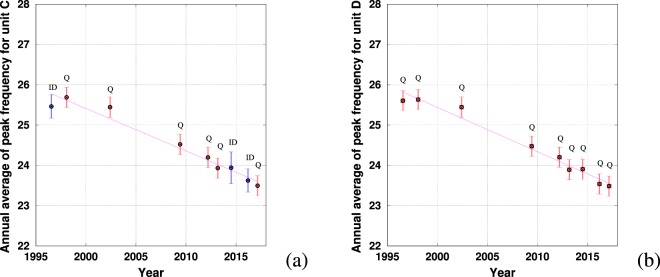
Temporal evolution of the peak frequency of units *C* (**a**) and *D* (**b**), computed by a Fast Fourier transform of the signal. The error bars color code is: red when quantification error is the greatest source of error (Q), blue when intrinsic dispersion is the greatest source of error (ID). A letter corresponding to the largest source of error is given on top of each error bar. The line is the linear interpolation by least-square error of the points displayed in the graph (the coefficient of determination ${R}^{2}$ being close to 0.95).

**Figure 6:**
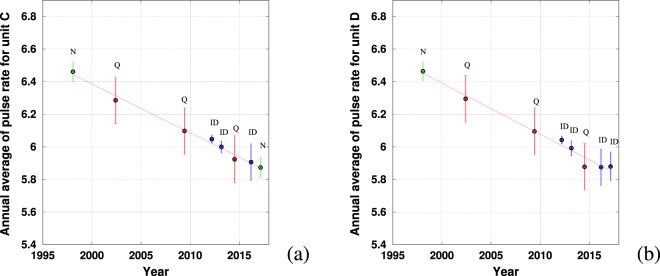
Evolution of the pulse rate of units *C* (**a**) and *D* (**b**). Error bars are red when quantification error is the greatest source of error (Q), green when error due to the noise is the greatest source of error (N), blue when intrinsic dispersion is the greatest source of error (ID). A letter corresponding to the largest source of error is given on top of each error bar. The line is the linear interpolation by least-square error of the points displayed in the graph (the coefficient of determination ${R}^{2}$ being close to 0.98).

The measurement of peak frequency by means of a FFT was robust despite increasing background noise: for all levels of noise measured, the main error in the measurement comes from the quantification error. The main noises in the recordings were: short-duration (less than 1 s) low frequency (less than 30–40 Hz) sounds that are especially strong in bad weather and long tonal sounds from ship motors. As the FFT is a linear process and signal to noise ratios were sufficiently high, these sounds did not prevent us from accurately measuring peak frequency. As for the measurement of the pulse rate, these noises seemed to have a higher influence on measurement, probably given that auto-correlation is a non-linear process. By performing several measurements of ${f}_{pulse}$ for different noise levels, we estimated the uncertainties, which were on the order of 0.05 Hz or less, and shown as error bars in Figs. 5 and 6.

#### Intrinsic dispersion of frequencies

In theory, intrinsic dispersion is due to the difference between two different sounds emitted by two different whales; two different sounds emitted by the same whale; or two sounds emitted by the same whale but affected differently by propagation effects. For example, in the latter case, the Doppler effect changes the received frequency by ${f}_{received}=\frac{1}{1-v/c}\times {f}_{emitted}$ where *c* is the sound speed and *v* is the radial speed of the whale relative to the recording device. For a typical value of $v/c\simeq \mathrm{1/1000}$, the difference in the frequency estimation is of the order of 0.1%. That is, for a frequency of 25 Hz, a difference of 0.025 Hz is obtained (see^27^ for a detailed analysis). Dispersion uniquely caused by sound production (the former two cases) is extremely difficult to estimate and seems very small^28^.

To estimate the intrinsic dispersion, regardless of its cause, we computed the standard deviation of results obtained for each year for ${f}_{peak}$ and ${f}_{pulse}$. This standard deviation can also be affected by the two precedent sources of uncertainty (background noise and quantification of measurements). These different causes of uncertainty can be separated theoretically. In the data, they are usually not separable. However, in some instances they appear clearly, as for instance when standard deviation is zero due to quantification errors (quantification errors are then masking the intrinsic dispersion).

#### Representation of uncertainties

 The quantification error, the error due to noise and the standard deviation of measured values were computed for each year, and the greatest of the three values were selected to represent errors graphically in Figs. 5 and 6.

## Results

### Application to SEP2

Despite having measured peak and pulse frequencies for four units of 436 SEP2 phrases, these measures for units *A* and *B* did not give precise results since these units usually have lower SNR and are somewhat modulated in frequency (see Fig. 1). Consequently, only units *C* and *D* are analyzed in the following sections.

#### Shift in peak frequency

A clear decrease in peak frequency for the two units *C* and *D* between 1996 and 2017 is evident from Fig. 5. As found for other blue whale song types^4^, the shift in frequency appears to be linear over time.

The peak frequency of unit *C* dropped from 25.8 ± 0.25 Hz in 1996 to 23.6 ± 0.25 Hz in 2017, an average decrease of 0.10 ± 0.03 Hz per year. For unit *D*, peak frequency dropped from 25.8 ± 0.25 Hz in 1996 to 23.5 ± 0.25 Hz in 2017, resulting in a decrease of 0.11 ± 0.03 Hz per year. The decline rates of these two frequencies are not significantly different. The coefficient of determination ${R}^{2}$ is 0.95 in both cases. For almost all years, the main source of uncertainty was quantification error. This means that this method has reached its intrinsic limit of precision for analyzing this type of sound.

Data from the same site on different years are consistent with the general trend (such as data from eastern tropical Pacific in 1996 and 2002, data form the CTBT station in 2007 and 2014 and data from the Corcovado Gulf in 2012, 2013 and 2016): transmission loss and propagation could affect each frequency peak’s intensity but should not shift the general spectrum.

#### Shift in pulse rate

The pulse rate for SEP2 blue whale song type over 20 years (1997–2017) is shown in Fig. 6. The results for 1996 were not plotted because auto-correlation methods produced unreasonable values due to the very low sample rate of these recordings (${f}_{s}=100$ Hz). In general, the relative error for pulse rate measurements was higher than for peak frequency measurements, although quantification errors could be reduced (typically with a higher sample rate of recording) for pulse rate measurement but not for peak frequency. Recordings with a high sample rate and high SNR usually had lower associated uncertainty (eg. years 2012 and 2013). Moreover, calculating an average reduces the uncertainty when error is introduced due to noise or intrinsic dispersion, but it usually does not reduce the error due to quantification.

The decrease also appears linear for the pulse rate. This is consistent with a harmonic signal and the fixed ratio $\frac{{f}_{peak}}{{f}_{pulse}}$. The pulse rate of unit *C* dropped from 6.44 ± 0.06 Hz in 1997 to 5.87 ± 0.10 Hz in 2017, an average decrease of 0.03 ± 0.01 Hz per year. For unit *D*, the pulse frequency dropped from 6.45 ± 0.06 Hz in 1997 to 5.87 ± 0.10 Hz in 2017, resulting in a decrease of 0.03 ± 0.01 Hz per year. The coefficient of determination ${R}^{2}$ is 0.98 in both cases.

### Application to SEP1

**Figure 7:**
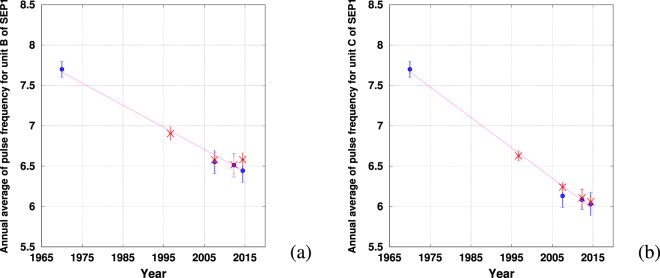
Pulse rate decrease in SEP1 phrases units. In blue (circles) the measures of pulse rate by mean of a summed auto-correlation method except the value of 1970 which is taken from literature^1^. In red (crosses), the points are computed by estimation of the frequency peak (by a FFT) divided by 3 and 4 for units *B* and *C* respectively ((**a**) and (**b**)). The errors bars are computed as for SEP2. The line is the linear interpolation by least-square error of the points displayed in the graph.

First, we checked if SEP1 units were harmonic by computing the values of ${f}_{peak}$ and ${f}_{pulse}$ for forty SEP1 phrases with high SNR selected from the 2012 and 2014 data sets following the method presented in^20^. ${f}_{pulse}$ was measured by an auto-correlation of the envelope of the signal. We found that $\frac{{f}_{peak}}{{f}_{pulse}}=6.23\pm 0.50$ for unit *A*, $3.06\pm 0.09$ for unit *B* and $4.07\pm 0.09$ for unit *C*. We thus assumed that the peak frequency of units *B* and *C* is an integer multiple of the pulse rate (as for units of SEP2) and therefore SEP1 units *B* and *C* are also harmonic. We then measured both the pulse rates (by summed auto-correlation of the signal) and peak frequencies of 107 high SNR units *B* and *C* of SEP1 selected from four years of data (see Table 1). We also included the value of the pulse rate for 1970 given in the literature^1^. Access to the precise values of peak frequencies for 1970 were unavailable as peak frequencies are given only at a precision of 1/3 octave. For 1996, the low sampling rate (100 Hz) did not enable us to make a precise measure of the pulse rate. Considering that $\frac{{f}_{peak}}{{f}_{pulse}}=3$ for unit *B* and $\frac{{f}_{peak}}{{f}_{pulse}}=4$ for unit *C*, we estimated the long term decline of ${f}_{pulse}$ combining both methods (see Fig. 7).

The decrease is clearly linear for pulse rate of units *B* and *C* and is of $0.029\pm 0.005$ Hz/year and $0.037\pm 0.005$ Hz/year respectively, which compares well with SEP2 (unit *C*: $0.03\pm 0.01$ Hz/year and unit *D*: $0.03\pm 0.01$ Hz/year). Interestingly, the two units *B* and *C* of the SEP1 song types are not decreasing at the same rate, despite these two units being quite similar in term of frequencies in 1970^1^. Since then, the two fundamental frequencies have decreased at a different rate and each unit currently appears quite different in the time-frequency representation (Fig. 1). Although SEP1 and SEP2 have similar time and frequency characteristics, and appear to occur with the same temporal distribution^17^, it remains unclear whether these song types indicate the presence of one or two acoustic groups.

## Discussion and conclusion

### Comparison between the methods

We observed a linear frequency shift in both SEP1 and SEP2 song types that is very similar to other blue whale song frequency shifts. We note that depending on the data, either peak frequency or pulse rate measurements give similar results. Error analyses show that pulse rate measurement with summed auto-correlation method has the best precision for data with a high sample rate (higher than 400 Hz). In cases where occurrences of a song type are scarce and several data sets are used, the combination of these two methods can give a better understanding of the trend in frequency shift. Nevertheless, we urge caution in the interpretation of the long-term analysis of SEP1 frequencies due to the small number of SEP1 occurrences analyzed (only 5 data points).

Future studies of frequency trends of song types of baleen whales should measure both peak frequency and pulse rate when it is possible. A precise value of pulse rate can be obtained whenever the signal is harmonic, the sampling rate and the SNR are high enough. And it is especially relevant to measure pulse rate when the sound is low frequency and not long compared to the pulse duration, as in most of pulsed blue whale song types.

### Comparison with frequency shifts in other regional song types

This study mainly compares two methods of measuring frequency shift and their respective advantages. However, having measured the trend in frequency shift of SEP1 and SEP2 songs over an extended time duration, our results align with frequency shifts in blue whale song measured elsewhere in the world. Comparison of our results were carried out against other frequency shifts and peak frequencies that have been reported in published papers (see Table 2). However, comparisons of pulse rates are not relevant in several song types that do not have pulsed units. Nevertheless, for each pulsed unit of each blue whale song type, a clarification of the link between the fundamental frequency and the pulse rate could enable us to better understand each song type, its production and to realize better measures of frequency trends.

**Table 2:** Comparison of our results to other works.

Song type	Period studied	Frequency (Hz)	Decrease in % of 2000’s value/year
Northeast Pacific *(a)*	1963–2008	21.9 to 15.2	0.91
Southwest Pacific *(a,d)*	1964–2013	25.3 to 17.5	0.81
Northwest Pacific *(a)*	1968–2001	23.0 to 17.9	0.86
North Atlantic *(a)*	1959–2004	23.0 to 17.6	0.66
Southern Ocean *(a,c,e,f)*	1995–2014	28.5 to 25.8	0.50
North Indian (unit 2) *(g)*	2002–2012	61.5 to 59.8	0.27
North Indian (unit 3) *(a,f,g)*	1984–2013	116 to 99.5	0.51
Southeast Indian (unit 2) *(b)*	2002–2010	72.5 to 69.5	0.51
Southeast Indian (unit 3) *(a)*	1993–2000	19.5 to 19.0	0.38
West Indian *(f)*	2007–2015	34.7 to 33.7	0.35
Southeast Pacific 1 (unit *B*)	1970–2014	23.1 to 19.3	0.43
Southeast Pacific 1 (unit *C*)	1970–2014	30.8 to 24.3	0.58
Southeast Pacific 2 (unit *C*)	1996–2017	25.8 to 23.6	0.44
Southeast Pacific 2 (unit *D*)	1996–2017	25.8 to 23.5	0.45

*(a)* McDonald *et al*.^4^ *(b)* Gavrilov *et al*.^9^ *(c)* Gavrilov *et al*.^5^ *(d)* Miller *et al*.^8^ *(e)* Leroy *et al*.^29^ *(f)* Leroy *et al*.^7^ *(g)* Miksis-Olds *et al*.^10^.

For other regional song types, the values of the peak frequencies are very different (many are around 20 Hz, but others are around 30 Hz and, in the north of Indian Ocean, the peak frequency is near 100 Hz). Thus, to compare different data, we computed the absolute values of the average decrease of the peak frequency during one year as % of the value estimated in a reference year. We chose the year 2000 as a reference year and calculated, for each song type, the mean value of the peak frequency during this year, by linear interpolation. The results are presented in Table 2.

The frequency decrease observed here in SEP2 song types is comparable to that of other song types (Table 2). Interestingly, all song types from the north and west Pacific Ocean have a greater decrease than songs from other oceans, while song types from the Indian Ocean have a smaller decrease than in other oceans.

Two different decreases are given for the southeast and north Indian ocean because two different units of the same song type have been measured. Recently, the last two units of the north Indian song type has been proved to decrease at different rates as in our study about SEP1 units B and C^10^. Studies have shown that several blue whale song types have intra-annual variations^5,8,7^. In our case we do not have such temporal precision and we cannot see these seasonal changes, which are masked by our error bars in Figs. 5 and 6.

### Pulse rate and peak frequency decrease in other species

Fin whales emit song types composed of short low frequency sounds around 20 Hz usually named “pulses”^30^. These sounds are repeated at a nearly constant rate which is also called “pulse rate” in the literature. A joint decrease of the pulse rate and peak frequency, at a different rate, has been recently noticed for fin whales songs in the northeast Pacific Ocean^12^. Decrease in peak frequency of fin whales calls in the Indian Ocean have been described by^7^. Decrease of fin whales pulse rates have also been described in north Atlantic Ocean^31^ and in northeast Pacific Ocean^32^. A frequency decrease has been found in an unidentified baleen whale “spot call”^6^, with sudden increase of peak frequency after some years of constant decrease. This unidentified baleen whale may be a southern right whale^6^ or a blue whale^13^.

This similarity between blue whales and other species’ sound emissions trends (pulse rate and frequency decrease over years) has to be addressed, since it is undoubtedly part of the worldwide question of whale song frequency decline.

A recent study^33^ proves that southeastern Indian Ocean blue whale song undergo another type of long term variations: the inter-song interval tends to increase resulting in a decrease of the phrase frequency (where phrase frequency can be defined as the number of phrases per second). This phrase frequency may not be linked to peak frequency and pulse rate in a simple way and its changes have to be studied for other song types. However, the global downward trend of most of the frequencies linked to whale song production needs to be addressed as a whole by the research community.

### Hypothesis on the reasons of the frequency shift

Several reasons were proposed for the frequency shift in blue whale song types, in the first paper about this phenomenon^4^: increase in body size post whaling, changes in anthropogenic noise and global climate change and the resulting ocean acidification were analyzed and ruled out as being too small effects to account for the shift. In the case of anthropogenic noise, the global trend is not the same worldwide, parts of the Indian Ocean undergoing a decrease of the global noise in low frequencies in the last decade^7^. Biological interference between blue whale songs and other biological sounds was also proposed^4^ but discarded later^10^.

The population recovery from whaling has been presented as the most probable reason for the frequency shift^4^: an increase in the density would result in lower amplitude vocalizations (because animals were no longer so far apart) which is hypothesized to allow lower-frequency vocalizations assuming that there is a trade-off between pitch and amplitude. However, in the last few decades, the songs of Antarctic blue whales have increased their SNR year after year^5^ and the songs in the north Indian Ocean seem to have a stable SNR^10^. For bowhead whales also, the minimum frequency of their calls has decreased for seven years whilst the source level of the calls has increased^11^. This is in contradiction with the mechanism proposed in^4^. In addition, two units of the north Indian Ocean have a frequency decrease at two different rates^10^, and the authors of the study claim that a change in the loudness of the song would likely result in the same rate of change for the two units. In our study, SEP2 song type has an identical trend for units *C* and *D* while the trend seems to be rather different for SEP1 units *B* and *C* which were of similar frequencies in the 1970’s. The precise function of the blue whale song and a fortiori of each of its units still remains unclear. However, the decadal change they undergo is probably not a mechanical response to an external factor.

Another cause that has been proposed is a change in the depth at which vocalizations are produced that could explain the frequency shift^9^. However, a recent study using recording tags^34^ seems to indicate that the depth of song production of the northeast Pacific blue whale can be variable up to a factor of two over very short time spans (a few minutes). If depth was a factor, the frequency content of the songs would vary strongly which is in contradiction with the identified stability, as shown by the very low standard deviation of the frequency components of the songs in our data and other studies^28^. Indeed, compared to other whales songs such as humpback whales’ (*Megaptera novaeangliae*)^35^ or bowhead whales’ (*Balaena mysticetus*)^36^ all blue whales song types are very repetitive songs with almost no changes in their structure.

A long-term combination of two contradictory effects could be a better way to explain the phenomenon of frequency decrease. Cultural conformity and sexual selection are also proposed as factors in^4^. On the one hand, there is an advantage for the population to be able to sing at exactly the same frequency^28^. For example, a female whale could know, using the Doppler effect, if she is approaching or going away from a male over large distances. On the other hand, a low frequency in a male’s song has been shown to be an advantage in sexual competition in several species, because it indicates a larger and more powerful singer^37^. The interplay between sexual competition (driving whales to sing at lower frequencies) and cultural conformism (based on the advantage for the whole population to vocalize with the same frequencies) could provide an explanation for the slow downward trend of a song whose main characteristic is its overall stability. In this context, the study of vagrant whales, acoustically isolated from their traditional population and migration paths, could help to understand the dynamics of this possible pressure.

Identification of decreasing frequency shifts across all blue whale populations could lead to a better understanding of poorly known blue whale behaviour. An understanding of the role played by the song in advancing and measuring blue whale population recovery is crucial in the context of increasing anthropogenic ocean noise.
